# Low bone mineral density is associated with the onset of spontaneous osteonecrosis of the knee

**DOI:** 10.3109/17453674.2012.684139

**Published:** 2012-06-04

**Authors:** Yasushi Akamatsu, Naoto Mitsugi, Takeshi Hayashi, Hideo Kobayashi, Tomoyuki Saito

**Affiliations:** ^1^Department of Orthopaedic Surgery, Yokohama City University Medical Center; ^2^Department of Orthopaedic Surgery, Yokohama City University School of Medicine, Yokohama City, Kanagawa, Japan.; Correspondence: akamatsu@yokohama-cu.ac.jp

## Abstract

**Background and purpose:**

The primary event preceding the onset of symptoms in spontaneous osteonecrosis in the medial femoral condyle (SONK) may be a subchondral insufficiency fracture, which may be associated with underlying low bone mineral density (BMD). However, the pathogenesis of SONK is considered to be multifactorial. Women over 60 years of age tend to have higher incidence of SONK and low BMD. We investigated whether there may be an association between low BMD and SONK in women who are more than 60 years old.

**Methods:**

We compared the BMD of 26 women with SONK within 3 months after the onset of symptoms to that of 26 control women with medial knee osteoarthritis (OA). All the SONK patients had typical clinical presentations and met specified criteria on MRI. The BMDs measured at the lumbar spine, ipsilateral femoral neck, and knee condyles and the ratios of medial condyle BMD to lateral condyle BMD (medial-lateral ratios) in the femur and tibia were compared between the two groups. The medial-lateral ratios were used as parameters for comparisons of the BMDs at both condyles.

**Results:**

The mean femoral neck, lateral femoral condyle, and lateral tibial condyle BMDs were between x% and y% lower in the SONK patients than in the OA patients (p < 0.001). The mean femoral and tibial medial-lateral ratios were statistically significantly higher in the SONK patients than in the OA patients.

**Interpretation:**

A proportion of women over 60 years of age have low BMD that progresses rapidly after menopause and can precipitate a microfracture. These findings support the subchondral insufficiency fracture theory for the onset of SONK based on low BMD.

The pathogenesis of spontaneous osteonecrosis of the medial femoral condyle (SONK) remains unclear, although a primary vascular insult and trauma are widely accepted as common causes. However, the pathogenesis of SONK is probably multifactorial (Zanetti et al. 2003, Robertson et al. 2009). Thus, it may be difficult to explain SONK for all ages and both sexes based on a single factor.

Lotke et al. (1977) first suggested a connection between the onset of SONK and subchondral fracture, which was supported by later reports based on MRI and pathological findings (Lecouvet et al. 1998, Yamamoto and Bullough 2000, Takeda et al. 2008). A subchondral insufficiency fracture may result from underlying osteoporosis (Yamamoto and Bullough 2000). This is consistent with a history of sudden onset of pain without a traumatic event (Ahlbäck et al. 1968). In other cases, obesity (Zanetti et al. 2003), overlying degenerative cartilage changes, meniscal tears (Ahlbäck et al. 1968), and meniscal injury (Robertson et al. 2009) may cause increased mechanical loading in the affected condyle, resulting in subchondral fracture.

One study using high-resolution quantitative computed tomography revealed that osteopenia and osteoporosis could be detected in two-thirds of patients with SONK diagnosed by MRI (Zanetti et al. 2003). These observations suggest that some cases of SONK are induced by subchondral insufficiency fracture that may be associated with an underlying low BMD. The incidence of SONK is more common in women than in men, and most of the patients are over 60 years of age (Lotke et al. 1977). A proportion of women older than 60 years have low BMD that progresses rapidly after menopause. Therefore, investigation of women older than 60 years—who may have a common factor—may be useful in clarifying the pathogenesis of SONK. In addition, because previous papers (Houpt et al. 1983, Mears et al. 2009, Robertson et al. 2009) have suggested the presence of preexisting knee osteoarthritis (OA) in patients with SONK, we compared patients with SONK and patients with medial knee OA. We assessed the relationship between recent onset of SONK and low BMD at various locations. We also compared the size of lesions by MRI in our SONK patients with those in previous reports.

## Patients and methods

### Subjects

**Figure 1.**
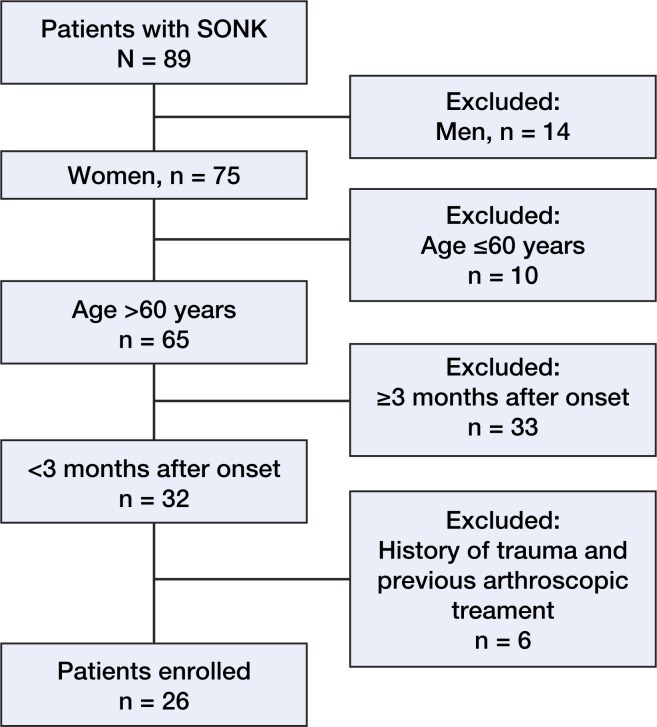
Flow diagram for identifying patients with SONK who were eligible.

**Figure 2.**
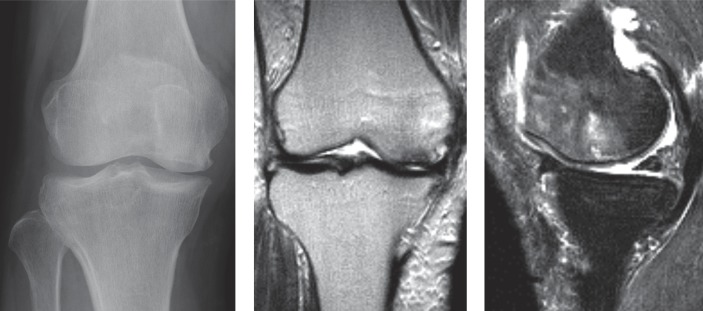
A. An AP radiograph from a 74-year-old woman, who had had sudden onset of right knee pain 7 weeks previously, showing a radiolucent oval lesion in the medial femoral condyle. The patient was classified as being at stage 2 of SONK and Kellgren-Lawrence grade 3. B. A coronal T2-weighted MRI showed an area of low signal intensity. C. A sagittal T2-weighted MRI with fat suppression showed subchondral changes and extensive bone marrow edema

**Figure 3.**
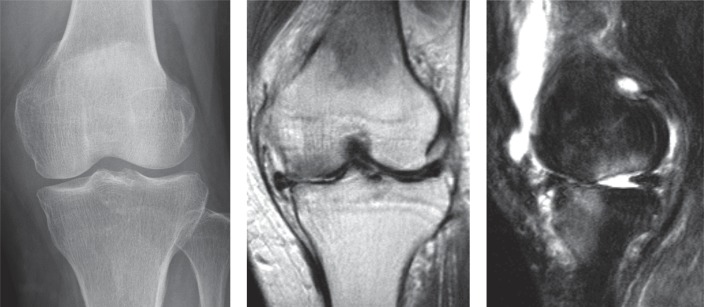
A. An AP radiograph from a 65-year-old woman, who had had sudden onset of left knee pain 10 weeks previously, showing no lesions in the medial femoral condyle. The patient was classified as being at stage 1 of SONK and Kellgren-Lawrence Grade 1. B. A coronal T2-weighted MRI showed an area of low signal intensity. C. A sagittal T2-weighted MRI with fat suppression showed subchondral changes and bone marrow edema

Between April 2005 and March 2009, we treated 89 consecutive patients with SONK. To target women over 60 years, we excluded 14 men and 10 women who were aged 60 years or less. To minimize the influence of disuse osteoporosis, women with SONK were only enrolled if no more than 3 months had elapsed between the onset of SONK and the time of the BMD measurements. Consequently, of the 65 patients remaining, we excluded 33 women because more than 3 months had elapsed since the onset of symptoms. In addition, we excluded 4 women for having a history of trauma and 2 women for previous arthroscopic treatment. None of the patients with SONK who were included in the study had corticosteroid injections, oral corticosteroid medication, or alcohol abuse. 26 patients remained (Figure 1). All patients were examined by radiography of the knee, knee MRI (Figures 2 and 3), and dual X-ray absorptiometry examinations of the lumbar spine, proximal femur, and knee condyles.

The mean delay between the onset of SONK and its diagnosis based on MRI and BMD measurements was 7 (2–12) weeks. All patients were initially managed nonoperatively. Worsening of knee pain in 15 patients led to surgical treatment mean 17 (6–38) weeks after the initial visit (in 8 patients: high tibial osteotomy; and in 7: a total or unicompartmental knee arthroplasty). Histological sections were obtained from the 15 patients who underwent surgery after a diagnosis of SONK. The diagnoses of the remaining 11 patients were based on the clinical presentation and imaging findings.

The clinical assessment was primarily based on the criteria established by Ahlbäck et al. (1968) and Lotke et al. (1977), comprising sudden onset of severe pain and localized tenderness over the medial femoral condyle.

Previous studies (Houpt et al. 1983, Mears et al. 2009, Robertson et al. 2009) have suggested the potential of preexisting knee OA in patients with SONK. In patients with medial knee OA, varus alignment can serve as a marker of disease severity or progression (Hunter et al. 2007). Thus, we formed a control group of 26 medial knee OA patients who were matched with respect to age, body mass index, and femorotibial angle (the OA group). These patients were considered to have knee OA if they had Kellgren-Lawrence grades (Kellgren and Lawrence 1957) of 2 or higher, and with a medial joint space narrowing of Ahlbäck grade I (Ahlbäck 1968) or lower. Thus, patients with obliteration of the medial joint space were excluded.

The Knee Society knee, pain, and function scores (Insall et al. 1989) were assigned at the first visit.

The study was approved by our institutional review board (number of approval: 1-10-2005-74), and all patients provided informed consent for participation in the study.

### Radiography

AP and lateral knee radiographs were taken with the patients standing. Limb alignment was expressed as the femorotibial angle obtained from the AP knee radiograph. The radiograph was also used to determine the stage of progression of SONK, which was classified into 4 stages (Koshino 1982): stage 1, normal radiographic appearance; stage 2, a radiolucent subchondral oval lesion or flattening of the convexity of the condyle, or both; stage 3, expansion of the radiolucent area surrounded by a sclerotic halo and a calcified plate; stage 4, secondary osteoarthritic changes. 6 knees with stage-1 disease had MRI confirmation and, on follow-up, had stage-2 disease or higher on plain radiographs within 1 year of the onset of disease.

### MRI

We used a 1.5-T GE Sigma Scanner (General Electric Medical Systems, Milwaukee, WI). Spin-echo pulse sequences were used exclusively for T1-weighted spin-echo images (repetition time, 610 ms; echo time, 22 ms) and T2-weighted spin-echo images (repetition time, 3,500 ms; echo time, 89 ms). Fat-suppressed images were also obtained. A slice thickness of 3 mm was chosen. The MRI diagnostic criteria for SONK included a discrete low-intensity area on the T1-weighted image and a corresponding low-intensity area with a surrounding high-intensity area, suggestive of bone marrow edema, on the T2-weighted and fat-suppressed images of the medial femoral condyle (Lotke and Ecker 1988, Lecouvet et al. 1998).

**Figure 4.**
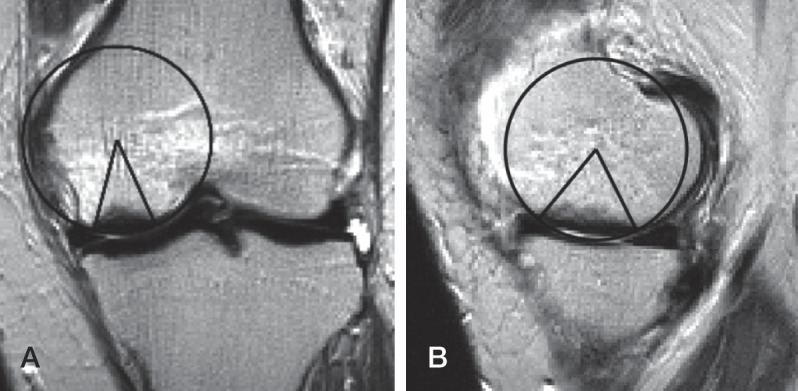
The necrotic angle (Mont et al. 2000) was measure in the sagittal plane (A) and the coronal plane (B). The 2 angles were summed to give the combined necrotic angle. In this case, the combined necrotic angle was 108° (40° + 68°).

All the SONK patients had findings in the weight-bearing area on MRI. In addition, we used the necrotic angle to measure the size of the epiphyseal lesion on MRI (Mont et al. 2000). The arcs of involvement of the subchondral lesion were measured using the center of the radius of the lesion, as measured from the epiphyseal scar in the sagittal and coronal planes (Figure 4). The 2 angles were summed to give the combined necrotic angle, which was used to assess the total lesions. Lesions of 150° or less were categorized as small, lesions of 151–249° were categorized as medium, and lesions of 250° or more were categorized as large.

### BMD

**Figure 5.**
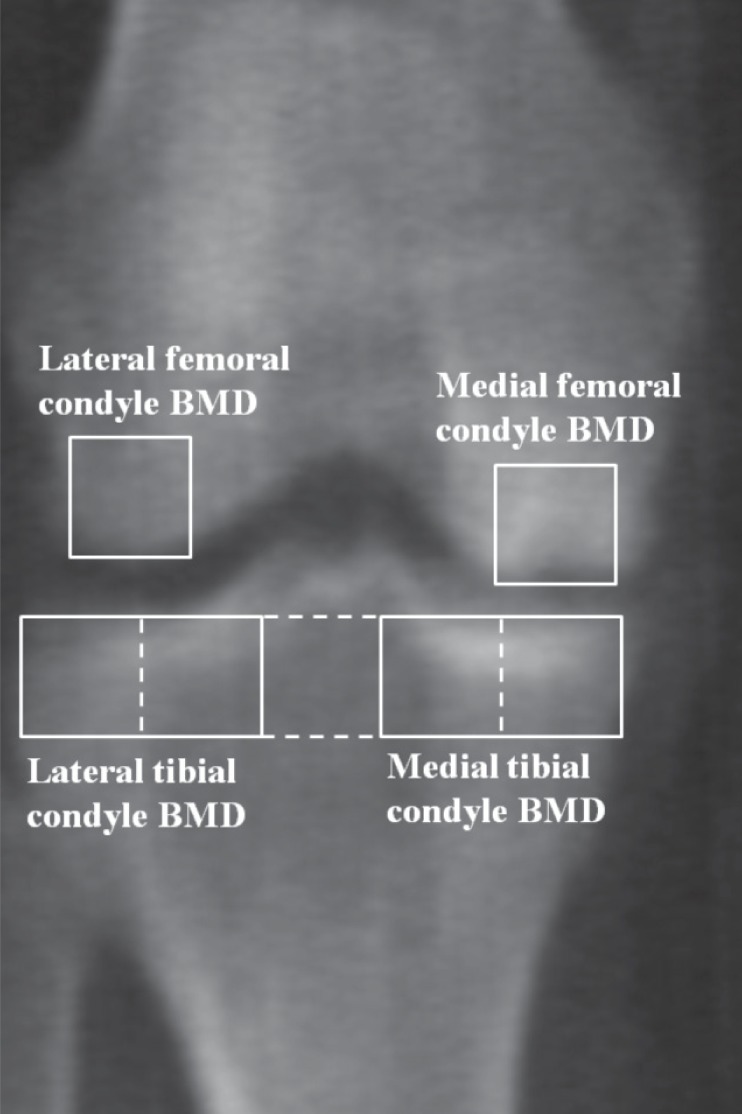
An AP dual X-ray absorptiometry image of the right knee of a 74-year-old woman 7 weeks after the onset of pain (the same patient as in Figure 2) showing a necrotic lesion surrounded by a sclerotic area in the medial femoral condyle. In the tibial condyles, five square regions of interest were marked on the frontal view. A line extending to the lateral and medial edges of the proximal tibia was divided into 5 equal lengths and 5 square regions of interest were marked underneath it. The medial tibial condyle BMDs in the 2 medial square regions of interest and the lateral tibial condyle BMDs in the 2 lateral square regions of interest were calculated for the tibia. In addition, the lateral and medial femoral condyle BMDs were calculated in square regions of interest of the same size as those on the tibial condyles located on a line passing through the tips of the medial and lateral condyles, with the midpoints of their distal sides at the points of contact.

We measured the BMD values at L2-L4 in the lumbar spine, the femoral neck, and the knee condyles using a QDR-4500 bone densitometer (Hologic Inc., Bedford, MA). We found no evidence of ipsilateral femoral neck BMD loss compared with the contralateral femoral neck BMD in either the SONK group or the OA group (p = 0.9 and p = 0.4, respectively). The BMD measurements for the knee condyles were performed with the patient in the supine position on the scanning table, with the knee flexed at an angle of 20° and the axis of the tibia parallel to the scanning table. In the tibial condyles, 5 square regions of interest were marked under a line on the proximal tibia. The medial tibial condyle BMDs in 2 medial square regions of interest and the lateral tibial condyle BMDs in 2 lateral square regions of interest were calculated for the tibia (Figure 5). In addition, we calculated the lateral and medial femoral condyle BMDs in square regions of interest of the same size as those on the proximal tibia marked on the femoral condyles. The ratios of the medial condyle BMD to the lateral condyle BMD (medial-lateral ratios) in the femur and tibia were used as parameters for comparisons of the BMDs at both condyles. Previous data have shown that this method is reliable (Akamatsu et al. 1997).

The patients were categorized according to the WHO definition (1994). A T-score of more than −1 was defined as normal, osteopenia was defined as a T-score of −1 to −2.49, and osteoporosis was defined as a T-score of −2.5 or less. In addition, low BMD was defined as a T score of −1 or less, which meant the sum of osteopenia and osteoporosis.

### Statistics

Data were expressed as the mean with 95% confidence interval (CI). Values were checked for normal distribution with the Shapiro-Wilk test. Differences between the groups were determined by Student’s t-test for continuous variables with normal distribution (age, height, weight, femorotibial angle, lumbar spine BMD, femoral neck BMD, lateral femoral condyle BMD, medial tibial condyle BMD, lateral tibial condyle BMD, and medial-lateral ratios); the Mann-Whitney test was used for continuous variables without normal distribution (BMI, Knee Society scores, and medial femoral condyle BMD); and the Pearson chi-square test (low BMD or normal based on the T scores at the lumbar spine) or Fisher’s exact probability test (low BMD or normal based on the T-scores at the femoral neck) was used for nominal variables. SPSS software version 17 was used for the statistical analyses. Values of p < 0.05 were considered significant. We performed a priori power analysis to ensure that the study was not underpowered. Since were no similar previous studies, we compared the BMDs between the two groups using a Cohen’s large effect size of 0.8 and a significance level of 0.05. We found that 80% power corresponded to a sample size of 26 subjects per group.

## Results

2 of the authors (YA, NM) measured the femorotibial angle on 135 knee radiographs and the interobserver interclass correlation coefficient was 0.996. In addition, the same authors classified the SONK lesions, and the kappa coefficient to determine the interobserver agreement of the radiographic staging was 0.88.

At entry into the study, the mean Knee Society knee, pain, and function scores were higher in the OA group than in the SONK group (Table 1).

**Table 1.** Patient characteristics. The values are given as mean (95% CI)

Variable	SONK	OA	95% CI	p-value
	(n = 26)	(n = 26)	for difference	
Age, years	72 (70–74)	71 (68–73)	–1.5 to 4.7	0.3 **^a^**
Height, cm	150 (147–152)	152 (149–154)	–5.2 to 1.5	0.3 **^a^**
Weight, kg	55 (52–59)	55 (52–58)	–4.0 to 4.5	0.9 **^a^**
Body mass index, kg/m^2^	25 (23–26)	24 (23–25)		0.4 **^b^**
Knee Society score, points				
Knee	53 (48–58)	72 (66–78)		< 0.001 **^b^**
Pain	20 (17–24)	35 (31–39)		< 0.001 **^b^**
Function	47 (40–53)	77 (68–86)		< 0.001 **^b^**

**^a^** Student’s t-test.

**^b^** Mann-Whitney test.

All knees with spontaneous osteonecrosis had femorotibial angles of more than 174°; the Kellgren and Lawrence grades were grade 1 in 5 knees, grade 2 in 19 knees, and grade 3 in 2 knees. In the OA group, the Kellgren and Lawrence grades were grade 2 in 8 knees and grades 3–4 in 18 knees. The radiographic stages of SONK at the time of diagnosis were stage 1 in 7 knees and stage 2 in 19 knees. The mean combined necrotic angle of the SONK group was 165°. Small lesions were noted in 7 knees and medium lesions were noted in 19 knees (Table 2).

**Table 2.** Imaging findings

Variable	SONK	OA	95% CI	p-value
	(n = 26)	(n = 26)	for difference	
Femorotibial angle (°)	180 (179–181)	179 (178–190)	–0.2 to 2.1	0.1 **^a^**
Kellgren-Lawrence grade (knees)				
Grade 1	5	0		
Grade 2	19	8		
Grade 3–4	2	18		
Radiographic stage (knees)				
Stage 1	7			
Stage 2	19			
Stage 3 to 4	0			
Combined necrotic angle (°)	165 (154–176)			
Lesion size (knees)				
Small	7			
Medium	19			
Large	0			

**^a^** Student’s t-test.

The mean femoral neck BMD was lower in the SONK group than in the OA group (CI for difference: –0.12 to –0.04; p < 0.001). All the knees in the SONK group had low BMDs at the femoral neck, and 20 of 26 knees in the OA group had low BMDs at the femoral neck (p = 0.02). However, there was no statistically significant difference in mean lumbar spine BMD between the 2 groups. The mean lateral femoral and tibial condyle BMDs were significantly lower in the SONK group than in the OA group (CI for difference: –0.19 to –0.07, p < 0.001; and CI for difference: –0.20 to –0.06, p < 0.001, respectively). However, the mean medial femoral and medial tibial condyle BMDs were similar between the two groups. The mean femoral and tibial medial-lateral ratios were significantly higher in the SONK group than in the OA group (CI for difference: 0.17–0.58, p = 0.001; and CI for difference: 0.01–0.34, p = 0.04, respectively) (Table3).

**Table 3.** BMD at various locations and medial-lateral ratios. The values are given as mean (95% CI)

Variable	SONK	OA	95% CI	p-value
	(n = 26)	(n = 26)	for difference	
Lumbar spine BMD, g/cm^2^	0.79 (0.73–0.84)	0.84 (0.79–0.89)	–0.13 to 0.02	0.2 **^b^**
Based on the T score at the lumbar spine				
Low BMD **^a^** / Normal BMD (knees)	20 / 6	20 / 6		1.0
Femoral neck BMD, g/cm^2^	0.54 (0.51–0.56)	0.62 (0.59–0.65)	–0.12 to –0.04	< 0.001 **^b^**
Based on the T score at the femoral neck				
Low BMD **^a^** / Normal BMD (knees)	26 / 0	20 / 6		0.01
Medial femoral condyle BMD, g/cm^2^	1.04 (0.96–1.12)	1.02 (0.95–1.09)		1.0 **^c^**
Lateral femoral condyle BMD, g/cm^2^	0.57 (0.53–0.61)	0.70 (0.65–0.75)	–0.19 to –0.07	< 0.001 **^b^**
Femoral medial-lateral ratio	1.86 (1.69–2.03)	1.48 (1.36–1.60)	0.17 to 0.58	0.001 **^b^**
Medial tibial condyle BMD, g/cm^2^	0.78 (0.69–0.86)	0.82 (0.75–0.89)	–0.15 to 0.04	0.2 **^b^**
Lateral tibial condyle BMD, g/cm^2^	0.52 (0.48–0.57)	0.65 (0.60–0.71)	–0.20 to –0.06	< 0.001 **^b^**
Tibial medial-lateral ratio	1.45 (1.31–1.59)	1.27 (1.18–1.36)	0.01 to 0.34	0.04 **^b^**

**^a^** Low BMD was defined as a T score of –1 or less.

**^b^** Student’s t-test.

**^c^** Mann-Whitney test.

## Discussion

We found that BMD of the femoral neck, lateral femoral condyle, and lateral tibial condyle were significantly lower in the SONK patients than in the OA patients. Also, the femoral and tibial medial-lateral ratios were significantly higher in the SONK patients than in the OA patients. Our findings support the subchondral insufficiency fracture theory for the onset of SONK from low BMD in women > 60 years of age.

The SONK group had a significantly lower BMD at the femoral neck than the OA group, and all the SONK patients had low BMDs at the femoral neck. In addition, similar to the case of the femoral neck, the SONK group had significantly lower BMDs at the lateral femoral and lateral tibial condyles than the OA group. The lateral condyle BMDs represent the BMD of the ipsilateral lower extremity in all of the knee condyles, as we found in a previous study (Akamatsu et al. 2009). These findings therefore suggest that recent onset of SONK has an association with low BMD in women over 60 years. The SONK group had lower BMD values at the femoral neck, but not at the lumbar spine, than the OA group. The hip is less affected by OA with age than the spine. Because lumbar spine osteophytes affect most subjects over 60 years and indicate false higher lumbar spine BMD values, diagnosis of osteoporosis in the elderly should be based on hip BMD (Liu et al. 1997). We therefore evaluated the BMD values at the femoral neck.

Abnormal medial-lateral ratio, which is a parameter for comparison of the medial condyle BMD with the lateral condyle BMD, in patients with knee OA is associated with increases in varus deformity (Akamatsu et al. 1997), bone marrow lesions, osteophytes, joint space narrowing, and sclerosis (Lo et al. 2006). We have found study on the comparison in the medial-lateral ratios between the SONK and the OA groups. The higher femoral medial-lateral ratio in the SONK group than in the OA group in our study corresponds to previous scintigraphic findings that the mean medial-lateral ratios of the distal femur plus proximal tibia in the early phase of SONK were higher than the ratios in knee OA (Muheim et al. 1970). We speculate these results show new bone formation accompanying the onset of SONK. We detected bone formation in the medial femoral condyle prior to the radiographic detection of the surrounding sclerotic halo.

Previous papers (Houpt et al. 1983, Mears et al. 2009, Robertson et al. 2009) have suggested the presence of preexisting knee OA in patients with SONK. It is likely that knee OA is common in women older than 60 years, and that most of our SONK patients had knee OA before the onset of osteonecrosis. We found Kellgren-Lawrence grades of 2 or higher in the knees of 21 of the 26 patients with SONK. This finding of preexisting knee OA in our SONK group is similar to published results (Houpt et al. 1983, Mears et al. 2009). Furthermore, overlying degenerative cartilage changes and medial meniscus or meniscal root injury (Ahlbäck et al. 1968, Robertson et al. 2009), which could be degenerative osteoarthritic changes, may weaken the load-bearing capacity (Narváez et al. 2003) and occur with increased loading at the medial femoral condyle, resulting in subchondral fracture. Thus, the pathogenesis of SONK may not only be related to low BMD but also to preexisting knee OA.

The degree of the combined necrotic angle on MRI correlates with the prognosis (Mont et al. 2000, Yates et al. 2007), with one report describing patients who had small combined necrotic angles of 150° or less and who were all clinically recovered at mean 5 months after the onset of disease (Yates et al. 2007). The mean combined necrotic angle in our SONK group was 165°. One reason for this difference may be related to the fact that our SONK patients all had lesions in the weight-bearing area, unlike in the previous study (Yates et al. 2007).

Our study design had several limitations. First, since we limited our study to subjects who presented within 3 months of the onset of clinical symptoms, only a small number of eligible patients were enrolled—and all cases were diagnosed with stage-1 or stage-2 disease using plain radiographs. The unremarkable radiographic changes delayed the diagnosis (Ahlbäck et al. 1968), and it was difficult to compile patients with SONK at the early stage after disease onset. Second, the lower Knee Society function score in the SONK group than in the OA group resulted from restricted activities of walking and climbing or descending stairs because of severe pain. However, we considered that limiting the duration to within 3 months of the onset of symptoms minimized the influence of disuse osteoporosis, because we found no evidence of differences between the bilateral femoral neck BMDs in the SONK group and the OA group at entry into our study. The distinctions between SONK and the osteonecrotic-like lesions found in knee OA using MRI were equivocal (Ahuja and Bullough 1978). Therefore, the criteria of typical clinical presentations (Ahlbäck et al. 1968, Lotke and Ecker 1988) and lesions in the weight-bearing area (Lotke and Ecker 1988) were essential for diagnosis of SONK in our study.

A recent report has indicated that the use of bisphosphonate prevents collapse in osteonecrosis of the femoral head (Lai et al. 2005). Future investigations should consider whether increasing the BMD, including the use of medications for osteoporosis, is an appropriate nonoperative approach to treatment of patients with SONK.
